# Toxic leadership and quality of work life: the moderating role of nurses’ agility

**DOI:** 10.1186/s12912-025-03776-5

**Published:** 2025-09-17

**Authors:** Ahmed Farghaly Tawfik, Ayman Mohamed El-Ashry, Amal Diab Ghanem Atalla, Shimaa Abd El-Fattah Mahgoub

**Affiliations:** 1 Nursing Administration, Faculty of Nursing, Beni-Suef University, Beni-Suef, Egypt; 2 Present Address: Psychiatric and Mental Health Nursing, Faculty of Nursing, Alexandria University, Alexandria, Egypt; 3 Nursing Administration, Faculty of Nursing, Alexandria University, Alexandria, Egypt; 4 Psychiatric and Mental Health Nursing, Department of Nursing, College of Applied Medical Science, Jouf University, Al-Qurayyat, Kingdom of Saudi Arabia

**Keywords:** Toxic leadership, Quality of work life, Nurses’ agility, Moderation

## Abstract

**Background:**

Toxic leadership undermines morale, job satisfaction, and retention among nurses. While previous studies have established its detrimental effects, the moderating role of nurses’ agility—a capacity for adaptation and resilience—remains underexplored, especially in the Egyptian healthcare context.

**Objective:**

This study examines the direct impact of toxic leadership on nurses’ quality of work life and explores whether nurses’ agility can mitigate these negative effects.

**Design and Methods:**

A cross-sectional, correlational design was used involving 265 full-time nurses across three hospitals in the Beni-Suef governorate, Egypt. Data collection instruments included the Toxic Leadership Scale, the Work-Related Quality of Life Scale, and the Workforce Agility Scale. Statistical analyses involved Pearson’s correlation, multiple linear regression, and moderation analysis via Hayes’ PROCESS macro.

**Results:**

Toxic leadership had a statistically significant negative impact on nurses’ quality of work life (*r* = -0.503, *p* < 0.001). Conversely, agility was positively associated with both toxic leadership (*r* = 0.159) and quality of work life (*r* = 0.425). Moderation analysis showed that nurses’ agility significantly buffered the negative effect of toxic leadership on quality of work life (β = 0.0049, *p* < 0.05).

**Conclusion:**

Toxic leadership erodes nurses’ quality of work life, but nurses with high agility experience less deterioration in work satisfaction and well-being. This suggests that agility operates as a protective factor.

**Implications for Nursing & Policy:**

Healthcare institutions must address toxic leadership through leadership development programs and should proactively enhance nurses’ agility through training in adaptability, stress management, and situational problem-solving. This dual strategy can improve nurse retention and ultimately, patient care outcomes.

**Patient or Public Contribution:**

Not applicable.

**Clinical trial number:**

Not applicable.

## Background

### Context and problem statement

Toxic leadership has emerged as a significant organizational threat in healthcare, where the cost of dysfunctional management extends beyond employee dissatisfaction to include patient safety risks, increased turnover, and diminished care quality [1, 2]. While the literature confirms the adverse effects of toxic leadership on nurses’ morale, commitment, and performance [3, 4], the factors that help buffer or neutralize these effects remain underexplored.

One promising yet under-researched construct is nurses’ agility, their ability to adapt, respond, and thrive in dynamic and high-pressure work environments. Despite its relevance to modern healthcare delivery, little empirical work has examined how agility might not just moderate, but significantly mitigate the negative impact of toxic leadership, particularly within Egyptian hospitals, where leadership training is limited and structural hierarchies are rigid [4, 5]. This potential for agility to act as a powerful buffer against toxic leadership is a source of hope and optimism in the healthcare management field.

This study addresses that gap by investigating how nurses’ agility influences the relationship between toxic leadership and their quality of work life (QWL). Framing this within Social Exchange Theory (SET), the study posits that when leadership becomes toxic, the reciprocal balance of workplace relationships is disrupted, resulting in stress and disengagement. However, nurses with high agility may be more equipped to navigate this imbalance and maintain psychological and professional stability [6, 7].

### Leadership dysfunction in healthcare

Historically, leadership in nursing was viewed through a positive lens, focusing on motivation, vision, and ethical stewardship [8]. However, recent scholarship has called attention to toxic leadership as a distinct and pervasive phenomenon that inflicts widespread harm across healthcare systems [3, 9, 10].

Toxic leadership is not merely ineffective leadership. It is marked by chronic behaviors such as abuse, narcissism, authoritarianism, self-promotion, and strategic unpredictability [9–11]. These leaders often prioritize personal power over team success, creating hostile and psychologically unsafe environments [1, 2]. In healthcare, this translates to diminished nurse engagement, strained team dynamics, and increased patient care errors [5, 11].

Studies by Griffin et al. [1] and Emblemsvåg [10] emphasized how toxic managers can drive out effective leaders, poison team culture, and erode organizational resilience. In Egyptian healthcare, Diab & Hassan [4] found strong associations between toxic leadership and reduced productivity, trust, and job satisfaction. Mahgob et al. [5] similarly noted an increase in counterproductive work behavior and intent to leave among nurses exposed to toxic behaviors.

### Quality of work life (QWL) and leadership impact

QWL is defined as the degree to which nurses’ work experiences satisfy important personal needs while enabling them to meet organizational goals [12]. High QWL is linked to increased performance, patient safety, and staff retention [13, 14]. However, it is highly sensitive to factors such as shift schedules, decision-making authority, workload, and—critically—leadership style [15, 16]. Multiple studies confirm that toxic leadership diminishes QWL by creating emotionally taxing environments, disrupting communication, and undermining trust [4, 16]. Conversely, supportive leadership improves engagement, emotional well-being, and perceived organizational justice [13, 16]. However, prior studies often treat QWL as a passive outcome, failing to explore how individual nurse characteristics—like adaptability or agility—can modulate this outcome under stress [6, 17].

### Workforce agility: a strategic buffer

Workforce agility refers to an individual’s ability to proactively adapt to change, learn quickly, and operate effectively in unpredictable environments [18, 19]. In healthcare, agile nurses are more likely to respond constructively to crises, take initiative, and maintain performance despite systemic dysfunction [4, 20, 21]. Cyfert et al. [20] and Sherehiy [22] argue that agility is not innate; it is cultivated through leadership support, continuous learning, and psychological empowerment. Agile nurses demonstrate better emotional regulation, innovation, and team collaboration—traits that may serve as buffers against toxic leadership [23–25]. Despite this potential, empirical evidence on agility as a moderator is sparse. While some literature suggests that agile employees are more resilient and less susceptible to managerial dysfunction [7, 26], few studies directly test this moderating effect, particularly in healthcare settings characterized by rigid hierarchies and limited managerial development [4, 5, 27].

### Research gap and study rationale

Prior work has been established: Toxic leadership impairs nurses’ QWL and increases turnover intentions [2, 4, 5]. Organizational support and emotional coping provide partial mitigation [28, 29]. Agility enhances adaptive behavior and job performance [19, 23, 25]. Yet, no studies in Egypt—or elsewhere—have empirically tested whether nurses’ agility can moderate the relationship between toxic leadership and QWL. This constitutes a critical gap, especially as healthcare systems seek resilient, adaptable workforces amidst post-pandemic instability. The novelty of our approach is sure to intrigue and pique the interest of our readers.

By exploring this triadic relationship, the present study adds conceptual and practical value to both the nursing leadership literature and the design of policy interventions. These findings are not just informative, but also crucial for the design of policy interventions that promote both healthier leadership and a more adaptable nursing workforce, making the audience feel engaged and interested in the study’s implications.

### Study objectives

1. To assess the relationship between toxic leadership and nurses’ quality of work life.
2. To examine whether nurses’ agility moderates the negative impact of toxic leadership on QWL.

### Hypotheses

- **H1**: Toxic leadership negatively affects nurses’ quality of work life.
- **H2**: Nurses’ agility moderates the relationship between toxic leadership and QWL, reducing its adverse effect.

## Method

### Research design

A correlational analytical research design was utilized to carry out this study following STROBE guidelines.

### Study setting

The study was conducted at three hospitals: Fever Hospital, Ophthalmology Hospital, and a university-affiliated to Beni-Suef University, all located in the Beni-Suef governorate of Egypt. These hospitals provide various healthcare services, including diagnostic, curative, and operative care.

### Subjects

The research focused primarily on nurses in the hospital’s inpatient care units. Using a convenience sampling method, eligible nurses meeting specific criteria were enrolled, which included (a) full-time nurses, (b) possessing a minimum of one year of experience in their clinical practice, and (c) those who provided informed consent for voluntary participation. The G*Power Windows 3.1.9.4 software determines the sample size required for a study, assuming a multiple linear regression with a moderate effect size, an alpha of 0.05, and a power of 0.95. Considering 13 variables—including five sociodemographic and eight scale-related dimensions, the minimum sample size was calculated to be 189 nurses. Considering that previous studies have reported dropout rates of 10% [29], the sample size was adjusted to a minimum of 208 nurses. In this study, 300 questionnaires were initially collected, and 265 valid questionnaires were retained, resulting in an effective response rate of 88.3%.

### Instruments

#### Sociodemographic characteristics

The researcher designed this part to collect data about the sociodemographic characteristics of study subjects, including age, gender, material status, qualification, and department.

### Toxic leadership scale

The Toxic Leadership Scale is a self-report questionnaire to evaluate nurse interns’ perceptions of toxic leadership. It was developed by Schmidt in 2008 [30] and consists of 30 items categorized into five primary dimensions: abusive supervision (7 items), authoritarian leadership (6 items), narcissism (5 items), self-promotion (5 items), and unpredictability (7 items). The internal consistency reliability, measured using Cronbach’s Alpha, was 0.979. Participants rate their level of involvement in job-crafting behaviors or thought processes on a Likert-type scale ranging from 1 (strongly disagree) to 6 (strongly agree). A higher total score indicates better job crafting, while a lower score indicates the opposite. Cronbach’s Alpha coefficient for this research was calculated to be 0.920.

### Quality of work life scale

The Quality of Work Life Scale, a comprehensive tool developed by Van Laar et al. in 2007 [31], assesses various factors related to both work and non-work domains. With 23 items across six factors, and an additional 24th item used as an outcome measure for assessing the reliability and validity of the items, it provides a thorough evaluation. Participants rate these items on a five-point Likert scale, with higher scores indicating higher levels of agreement. The total Quality of Work Life score, obtained by summing the scores from the six subscales, offers a holistic view. In the present research, Cronbach’s Alpha coefficient was calculated to be 0.905, further reinforcing the scale’s effectiveness.

### Workforce agility scale

The Workforce Agility Scale, a practical and relevant tool developed by Sherehiy in 2008 [22] and modified by [32], consists of 21 items divided into three categories: Proactive, Adaptive, and Flexible. It is designed to measure the degree of workforce agility among nurses, providing a tangible measure of their adaptability. A five-point Likert scale, with one denoting strongly disagree and five denoting strongly agree, is used to rate the subjects’ answers. A high score indicates higher nurses’ agility, and vice versa, making the scale’s results easy to interpret. The internal consistency reliability, measured using Cronbach’s Alpha, was found to be 0.868, further confirming its practicality. The researchers categorized nurses’ agility into three categories using median and standard deviation: high, moderate, and low, making the results easily applicable in real-world scenarios.

### Validity of the tools

Two professional translators translated the study tools into Arabic, followed by another round of translation back into English by two independent translators. A panel of five experts evaluated the accuracy and validity of the Arabic translation. This expert panel included professors from the nursing administration department who assessed the content and face validity of the translation. Their feedback was carefully analyzed and incorporated to ensure the study’s tools’ clarity, question types, and overall content validity. The integrity and accuracy of the translated tools were thoroughly reviewed to ensure their credibility.

### Ethical consideration

Under the reference number FMBSUREC-07072024/TAWFIK, the Research Ethics Committee of the Faculty of Medicine at Beni-Suef University in Egypt formally approved and granted authorization to perform the study on 7th July/2024. This study was conducted following the ethical principles outlined in the Declaration of Helsinki. Additionally, written informed permission was collected from study participants who voluntarily consented to participate in the research before their involvement in the study. The participants were informed about their right to withdraw without giving any reason and that the data collected would be kept confidential.

### Setting approval

To facilitate data collection, the managers of the previously mentioned Hospitals issued official approval before starting the study. Individual written consent was also obtained from each participant.

### Pilot study

Before commencing fieldwork and data gathering, a preliminary investigation involved 10% of the primary study participants (27 individuals). This initial study aimed to assess the comprehensibility of the questionnaire sheets and their pertinence to the research. Additionally, it aided in determining the time required to complete the data collection forms, which typically took 10 to 15 min to fill out. The participants involved in the pilot study were subsequently excluded from the primary study sample.

### Fieldwork

The study’s fieldwork was extended for two months. Data was collected from the beginning of July to the end of August 2024. After securing the official approval for the study, the researchers met the nurses and explained the study objectives to them. The information was gathered from nurses during their work hours according to their availability three days per week.

### Statistical design

The data was carefully reviewed for accuracy and consistency, then organized and inputted into Microsoft Excel 2019. After that, it was transferred to IBM SPSS Statistics version 25 for analysis. Descriptive statistics were used to present the study participants’ characteristics and variables, and mean scores and standard deviations were calculated for numerical values. A significance level of *p* < 0.05 was chosen, and a p-value of ≤ 0.001 was considered highly significant. Parametric tests was employed as the data met the assumption of normality, confirmed by the Kolmogorov-Smirnov test, Inter-Quartile Range analysis, and residual plots [33]. To manage outliers, the Winsorizing method was used in IBM SPSS, which replaces extreme values with the nearest acceptable value within the interquartile range [34]. multicollinearity was evaluated among variables, ensuring independent variables were not highly correlated. Tolerance values were above 0.1, and the Variance Inflation Factor (VIF) for all variables was below 1.5, indicating no significant multicollinearity. For moderation analysis, multiple linear regression was conducted. Additionally, Andrew Hayes Process Macro was used for data analysis based on ordinary least squares regression and bias-corrected bootstrapping. This approach provides more robust statistical power than other confidence interval analyses [35, 36].

## Results

As shown in Table 1, most nurses were female (72.5%), aged between 25 and 35 years, with a mean age of 27.64 years. Most were married (67.5%) and held a diploma-level qualification. Nearly half (46.6%) had between 5 and 10 years of clinical experience.

**Table 1:** Distribution of the study participants according to their characteristics (*n* = 265)

Variable	Category	Frequency	Percentage
Age	> 25	52	19.6
	25-	191	72.1
	35+	22	8.3
Mean + SD		27.64 + 4.08	
Gender	Male	73	27.5
	Female	192	72.5
Marital status	Single	125	47.2
	Married	107	40.4
	Divorced or Widowed	33	12.4
Qualifications	Diploma	125	47.2
	Bachelor	107	40.4
	Master	25	9.4
	PHD	8	3.0
Years of experience	1-	109	41.1
	5-	112	42.3
	10-	21	7.9
	15+	23	8.7
Total		265	100.0

Table 2 provides descriptive statistics and bivariate correlations among the study variables. Nurses reported moderate levels of toxic leadership and relatively high levels of both agility and quality of work life. A moderate negative correlation was observed between toxic leadership and QWL (*r* = -0.503, *p* < 0.001), and a positive correlation was found between agility and QWL (*r* = 0.425, *p* < 0.001). Interestingly, agility was also positively correlated with toxic leadership (*r* = 0.159, *p* = 0.010), suggesting potential adaptive responses under toxic conditions.

**Table 2:** Descriptive statistics and bivariate correlations of the study variables and their dimensions (*n* = 265)

		1	2	3	4	5	6	7	8	9	10	11	12	13	14	15	16	17
1	**Toxic Leadership**																	
2	Abusive supervision	0.787^**^																
3	Authoritarian Leadership	0.734^**^	0.710^**^															
4	Narcissism	0.695^**^	0.693^**^	0.682^**^														
5	Self-Promotion	0.727^**^	0.743^**^	0.743^**^	0.734^**^													
6	Unpredictability	0.715^**^	0.779^**^	0.648^**^	0.638^**^	0.801^**^												
**7**	**QWL**	**− 0.503** ^**^	**− 0.362** ^**^	**− 0.294** ^**^	**− 0.327** ^**^	**− 0.340** ^**^	**− 0.302** ^**^											
8	JCS	0.070	0.049	0.225^**^	0.120	0.085	0.214^**^	0.368^**^										
9	SAW	0.015	0.092	0.048	0.107	0.084	0.089	0.402^**^	0.476^**^									
10	WCS	-0.042	-0.001	0.039	-0.085	-0.067	-0.003	0.342^**^	0.462^**^	0.609^**^								
11	CAW	-0.005	0.018	0.168^**^	0.048	-0.002	0.001	0.400^**^	0.610^**^	0.485^**^	0.558^**^							
12	HWI	0.081	0.139^*^	0.217^**^	0.083	0.036	0.043	0.303^**^	0.514^**^	0.455^**^	0.442^**^	0.722^**^						
13	GWB	0.037	0.106	0.199^**^	0.084	0.010	0.047	0.402^**^	0.599^**^	0.550^**^	0.616^**^	0.698^**^	0.672^**^					
**14**	**Agility**	**0.159** ^**^	**0.149** ^*^	**0.180** ^**^	**0.156** ^*^	**0.081**	**0.122** ^*^	**0.425** ^**^	**0.344** ^**^	**0.329** ^**^	**0.236** ^**^	**0.307** ^**^	**0.281** ^**^	**0.310** ^**^				
15	Proactivity	0.094	0.124^*^	0.188^**^	0.254^**^	0.030	0.052	0.212^**^	0.452^**^	0.448^**^	0.289^**^	0.287^**^	0.283^**^	0.397^**^	0.478^**^			
16	Adaptability	0.216^**^	0.264^**^	0.295^**^	0.218^**^	0.076	0.170^**^	0.158^*^	0.390^**^	0.298^**^	0.257^**^	0.287^**^	0.407^**^	0.374^**^	0.516^**^	0.612^**^		
17	Resilience	0.081	0.141^*^	0.238^**^	0.029	-0.009	0.123^*^	0.316^**^	0.510^**^	0.355^**^	0.333^**^	0.404^**^	0.372^**^	0.489^**^	0.574^**^	0.596^**^	0.712^**^	
Mean ± SD		93.8 ± 25.2	19.96 ± 7.32	19.38 ± 5.25	17.52 ± 4.68	16.67 ± 4.46	23.20 ± 6.26	87.23 ± 13.9	23.46 ± 3.69	7.75 ± 1.37	11.62 ± 1.87	10.82 ± 2.34	10.77 ± 2.34	22.29 ± 3.81	84.80 ± 10.62	27.90 ± 4.11	27.78 ± 4.13	27.23 ± 4.03
Max. Score		150.0	35.00	30.00	25.00	25.00	35.00	115.0	30.00	10.00	15.00	15.00	15.00	30.0	105.0	35.0	35.0	35.0

**. Correlation is significant at the 0.01 level (2-tailed). *. Correlation is significant at the 0.05 level (2-tailed)

QWL = quality of work life, JSC = job career satisfaction, SAW = stress at work, WCS = work conditions, CAW = control at work, HWI = homework interface, GWB = general wellbeing

The regression model confirmed that toxic leadership significantly predicted QWL (Table 3, β = -0.417, *p* < 0.001), accounting for approximately 25.3% of the variance. Crucially, moderation analysis revealed a significant interaction effect between toxic leadership and agility on QWL (β = 0.0049, *p* = 0.028), supporting Hypothesis 2.

**Table 3:** Multiple linear regression for nurses’ quality of work life

Predictor	Estimate	SE	t	*p*	Stand. Estimate
(Constant)	113.277	2.859	39.626	0.000	
Toxic	− 0.278	0.029	-9.432	0.000	− 0.503
F	p		R	R Square	Adjusted R Square
88.972	0.000		0.503	0.253	0.250

Table 4 shows that the interaction between toxic leadership and nurses’ agility as a moderator on quality of work life is significant (Int_1, β = 0.0049, *p* < 0.05) when controlling for age, gender, marital status, qualification, and experience. The results of this moderation test indicated that nurses’ agility moderates the effect of toxic leadership on the quality of work life.

**Table 4:** Moderation test results PROCESS MACRO by Andrew F. HAYES, agility as a moderator in the relation between toxic leadership and nurses’ quality of work life

	β	se	t	*p*	LLCI	ULCI
Constant	104.5008	20.7772	5.0296	0.0000	63.5847	145.4169
Toxic leadership	− 0.7485	0.2073	-3.6115	0.0004	-1.1566	− 0.3403
Agility	0.1919	0.2302	0.8334	0.4054	− 0.2615	0.6453
Int_1	0.0049	0.0024	2.0869	0.0379	0.0003	0.0095
Age	− 0.1761	0.2085	− 0.8448	0.3990	− 0.5867	0.2344
Gender	− 0.6764	1.3706	− 0.4935	0.6221	-3.3755	2.0228
Marital status	0.8426	1.3679	0.6160	0.5384	-1.8511	3.5363
Qualification	-1.3432	0.8060	-1.6666	0.0968	-2.9304	0.2440
Experience	2.3370	0.9640	2.4244	0.0160	0.4387	4.2353
R^2^	0.544					
F	38.18					
DF1	8.00					
DF2	256.0					
P	0.000					
**Int_1**						
R^2^ change	0.008					
F	4.36					
p	0.037					

**Fig. 1:**
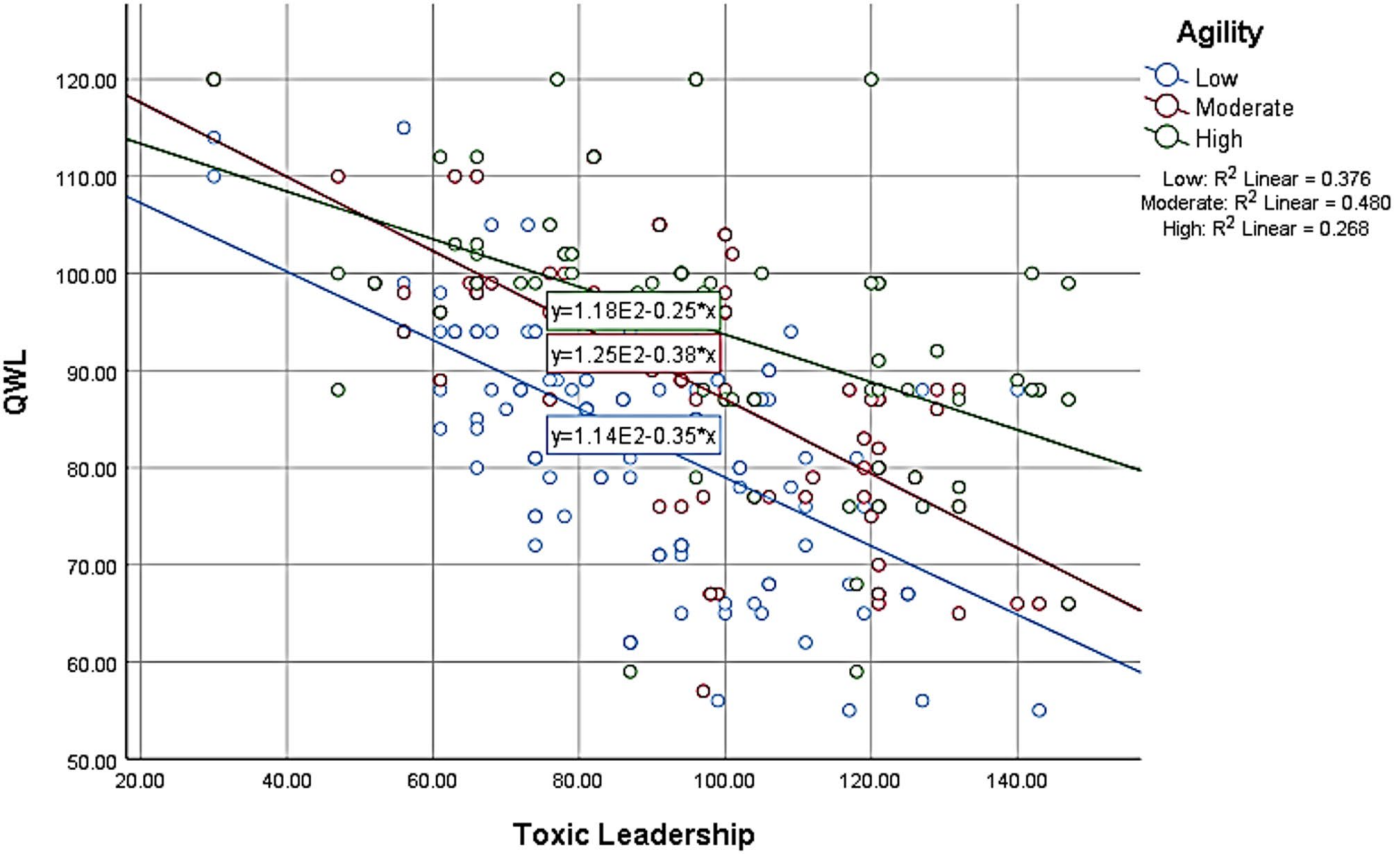
Moderation effect of agility the relation between toxic leadership and nurses’ quality of work life

As visualized in Fig. 1, the negative effect of toxic leadership on QWL was more pronounced among nurses with low agility. In contrast, those with high agility demonstrated greater resilience, maintaining higher QWL scores even under adverse leadership conditions.

## Discussion

This study examined the influence of toxic leadership on nurses’ quality of work life (QWL) and tested the moderating role of workforce agility. The results strongly supported both hypotheses and revealed three central insights: (1) toxic leadership has a substantial negative effect on QWL, (2) workforce agility is positively associated with QWL, and (3) agility significantly moderates the relationship between toxic leadership and QWL.

### Toxic leadership and QWL: a confirmed threat

Consistent with Hypothesis 1, toxic leadership was found to be a statistically significant negative predictor of nurses’ QWL (β = -0.417, *p* < 0.001). The correlation analysis further reinforced this, with a moderate inverse relationship (*r* = -0.503). These findings align with previous literature emphasizing the detrimental psychological and organizational effects of toxic leadership behaviors such as abuse, authoritarianism, and manipulation [2–4]. Nurses working under such conditions are likely to experience emotional exhaustion, lack of autonomy, and diminished morale—factors that collectively degrade their perceived quality of life at work [3, 4, 28].

Importantly, the regression model indicated that toxic leadership accounted for over 25% of the variance in QWL. This substantial effect size underscores the centrality of leadership behavior in shaping occupational well-being, more so than structural or demographic variables like age, gender, or years of experience, which were statistically controlled.

These results corroborate earlier Egyptian studies by Diab and Hassan [4] and Mahgob et al. [5], who reported that toxic supervisory styles were closely associated with high turnover intentions and disengagement among nurses. Globally, the pattern mirrors findings from Labrague’s [3] systematic review and Ofei et al. [11], both of which positioned toxic leadership as a systemic threat to health worker retention and patient safety.

### Workforce agility: a positive force

In contrast to the corrosive effects of toxic leadership, workforce agility emerged as a constructive force. It demonstrated a positive correlation with QWL (*r* = 0.425, *p* < 0.001), suggesting that nurses who perceive themselves as adaptable, proactive, and flexible tend to experience greater well-being, job satisfaction, and role clarity. This supports prior work by Sherehiy [22], Hussien Kamel et al. [25], and Nafei [21], who identified agility as a core competency for navigating modern healthcare complexities. This result also reflects the positive organizational psychology perspective, which suggests that individual-level capabilities like adaptability enhance not only performance but resilience and intrinsic motivation [7, 18, 19].

### Agility as a moderator: strategic implications

Most notably, Hypothesis 2 was supported by a significant interaction effect between toxic leadership and nurses’ agility (β = 0.0049, *p* = 0.028). The moderation analysis revealed that while toxic leadership negatively impacts QWL across the board, its effect is weaker among nurses with higher agility. In practical terms, agile nurses are better equipped to maintain well-being and psychological stability, even in hostile or dysfunctional work environments.

This insight is critical. While previous studies have shown that coping strategies or emotional intelligence may mediate or moderate workplace stress [28, 29], this study shows that agility functions as a forward-looking, structural capability, not merely a reactive defense mechanism. Agile nurses do not just survive toxic leadership; they adapt to it, find alternative paths of action, and preserve their professional functionality.

Interestingly, agility also showed a small but statistically significant positive correlation with toxic leadership (*r* = 0.159, *p* = 0.010). This suggests a nuanced dynamic: exposure to toxic environments may prompt some nurses to develop greater adaptability as a survival strategy. While potentially beneficial at the individual level, this also points to a troubling organizational reality—that agility may emerge not by design but by necessity, a red flag for health system leadership. These findings extend the work of Cyfert et al. [20], Ajgaonkar et al. [18], and Mohamed El-Sayed et al. [32], all of whom emphasized the need for leadership practices that cultivate, rather than inadvertently coerce, workforce agility [37].

### Strengths and limitations

This study offers a timely contribution to the growing literature on nursing leadership by addressing an underexplored question: how nurses’ agility moderates the relationship between toxic leadership and quality of work life. The integration of a theoretically grounded moderation model—supported by robust statistical methods and validated tools—adds methodological rigor to the study. The use of Hayes’ PROCESS macro, in particular, strengthens causal inference by incorporating bias-corrected bootstrapping and controlling for demographic covariates.

Another strength lies in the sample size and context. With 265 nurses across three hospitals in Egypt, the study captures diverse institutional realities and offers findings that are both statistically sound and practically relevant within similar healthcare systems. The use of well-established measurement scales for toxic leadership, agility, and QWL further enhances the reliability and validity of the findings.

However, some limitations must be acknowledged. First, the use of convenience sampling introduces potential selection bias and limits generalizability beyond the studied institutions. Second, the cross-sectional design restricts causal claims; while relationships are statistically significant, longitudinal research is needed to confirm directionality over time. Third, the study relies on self-reported data, which is vulnerable to social desirability, especially in hierarchical workplace cultures where toxic leadership may be underreported. Finally, the cultural specificity of the findings, while valuable, may not directly translate to healthcare settings with different organizational or leadership norms.

Despite these limitations, the study presents a meaningful step forward in examining how individual-level capacities like agility can function as strategic buffers against dysfunctional leadership, offering both theoretical advancement and actionable insights.

## Conclusion

This study provides robust empirical evidence that toxic leadership significantly undermines nurses’ quality of work life (QWL), while workforce agility serves as a moderating force capable of mitigating this impact. The statistical findings confirm that toxic leadership accounts for a meaningful portion of the variance in QWL, with nurses under toxic managers experiencing lower satisfaction, engagement, and psychological well-being. However, agility—defined as the ability to adapt, innovate, and recover in dynamic conditions—proved to be a strategic protective factor.

Nurses with higher levels of agility reported better QWL even under conditions of toxic leadership. This moderating effect highlights agility not just as a desirable trait, but as a functional necessity in today’s complex and often dysfunctional healthcare settings. It is not merely about adapting to change; it is about preserving personal and professional stability in the face of organizational adversity.

### Implications in nursing and health policy

This study offers valuable contributions for nursing practice and healthcare policy by highlighting both the detrimental effects of toxic leadership and the buffering role of workforce agility. The findings suggest that while nurses with higher adaptability are better equipped to maintain their quality of work life under toxic conditions, this should not be interpreted as tolerance for dysfunctional leadership. Instead, it emphasizes the urgency for a dual-intervention strategy: systematically reducing toxic leadership behaviors while simultaneously enhancing workforce agility. In clinical settings, agility should be recognized as a core nursing competency, on par with clinical expertise. Hospitals and health systems should implement structured agility training programs focusing on proactive behavior, situational decision-making, crisis response, and stress management, as supported by prior literature (e.g., Sherehiy, 2008 [22]; Nafei, 2018 [21]; Hussien Kamel et al., 2023 [25]; El-Sayed et al., 2022 [32]). Simulation-based learning and psychological empowerment approaches should also be adopted to cultivate adaptability and emotional resilience among nurses (Ajgaonkar et al., 2022 [18]; Cyfert et al., 2022 [20]).

At the same time, toxic leadership must be addressed through formal leadership development programs targeting emotional intelligence, ethical leadership, and communication—areas consistently identified as weak in toxic leadership profiles (Diab & Hassan, 2023 [4]; Ofei et al., 2023 [11]; Rothwell et al., 2023 [8]). Organizational interventions such as 360-degree feedback, leadership audits, and early-warning systems for high-turnover units can serve as mechanisms for early detection and correction. Moreover, psychological safety must become a measurable standard for leadership performance, not merely a cultural aspiration (Mahgob et al., 2024 [5]; Akter et al., 2018 [16]).

From a policy perspective, our findings argue for embedding leadership appraisal systems and workforce agility development into national nursing strategies and hospital accreditation frameworks. Regulatory bodies should require ongoing leadership training, and policy guidelines should explicitly identify and sanction toxic behaviors (Griffin et al., 2022 [1]; Wolor et al., 2022 [2]; Emblemsvåg, 2023 [10]). Simultaneously, structured national programs should be developed to build nurses’ agility and resilience as strategic assets for the health system (Mohamed Nagib et al., 2022 [27]; Mohamed El-Sayed et al., 2022 [32]).

## Data Availability

Data will be available on reasonable request from the corresponding author.
